# Aquatic Pseudomonads Inhibit Oomycete Plant Pathogens of *Glycine max*

**DOI:** 10.3389/fmicb.2018.01007

**Published:** 2018-05-29

**Authors:** Andrew Wagner, Stephen Norris, Payel Chatterjee, Paul F. Morris, Hans Wildschutte

**Affiliations:** Department of Biological Sciences, Bowling Green State University, Bowling Green, OH, United States

**Keywords:** *Pseudomonas*, oomycete, antagonistic, biocontrol, biosynthetic gene cluster

## Abstract

Seedling root rot of soybeans caused by the host-specific pathogen *Phytophthora sojae*, and a large number of *Pythium* species, is an economically important disease across the Midwest United States that negatively impacts soybean yields. Research on biocontrol strategies for crop pathogens has focused on compounds produced by microbes from soil, however, recent studies suggest that aquatic bacteria express distinct compounds that efficiently inhibit a wide range of pathogens. Based on these observations, we hypothesized that freshwater strains of pseudomonads might be producing novel antagonistic compounds that inhibit the growth of oomycetes. To test this prediction, we utilized a collection of 330 *Pseudomonas* strains isolated from soil and freshwater habitats, and determined their activity against a panel of five oomycetes: *Phytophthora sojae, Pythium heterothalicum, Pythium irregulare, Pythium sylvaticum*, and *Pythium ultimum*, all of which are pathogenic on soybeans. Among the bacterial strains, 118 exhibited antagonistic activity against at least one oomycete species, and 16 strains were inhibitory to all pathogens. Antagonistic activity toward oomycetes was significantly more common for aquatic isolates than for soil isolates. One water-derived strain, 06C 126, was predicted to express a siderophore and exhibited diverse antagonistic profiles when tested on nutrient rich and iron depleted media suggesting that more than one compound was produced that effectively inhibited oomycetes. These results support the concept that aquatic strains are an efficient source of compounds that inhibit pathogens. We outline a strategy to identify other strains that express unique compounds that may be useful biocontrol agents.

## Introduction

Soybean root rot caused by oomycete infections is a significant pathogen problem in soybeans fields across the Midwest. Because longer growing seasons directly correlate with increased soybean yields, early planting is the current preferred practice (Rowntree et al., 2013). However, cold soils, coupled with an unseasonably wet planting season, increases the risk of oomycete-mediated root rot seedling loss (Broders et al., 2007; Wei et al., 2011; Zitnick-Anderson and Nelson, 2015). Direct sampling of infected plants found that a wide range of pathogens were involved. A 2011 survey from 11 soybean producing states in the United States Midwest region, and the Canadian province of Ontario, resulted in the characterization of 2,378 oomycete isolates (Rojas et al., 2017). This collection included 51 *Pythium* spp., three *Phytophthora* spp., three *Phytopythium* spp., and one *Aphanomyces* sp. In the 2nd year of the survey, 54 *Pythium* spp., seven *Phytophthora* spp., six *Phytopythium* spp., and one *Phytigeton* sp. were recovered using a modified medium to enhance the recovery of slower growing *Phytophthora* strains. The most common isolates recovered from diseased plants were *Pythium sylvaticum* (16%) and *Pythium oopapillum* (13%) in 2011, and *Pythium sylvaticum* (14%) and *Pythium heterothallicum* (12%) in 2012. Some of these *Pythium* species such as *P. irregulare, P. sylvaticum, P. torulosum*, and *P. macrosporum* were cold adapted and most aggressive at soil temperatures below 20°C. Other species such as *P. ultimum* infect different hosts at temperatures ranging from 12 to 25°C (Wei et al., 2011), while *P. aphanidermatum* cause soybean seedling rot only at temperatures of 20–25°C (Rojas et al., 2017).

Numerous strategies have been employed to prevent infection from different oomycetes. The primary management tool to prevent disease from *P. sojae* has been the deployment of soybean varieties containing single genes that confer resistance (Tyler et al., 2006; Dorrance et al., 2007). Unfortunately, continued use of resistant soybean lines alone, results in the selection for oomycete races that are capable of overcoming all soybean resistance genes, and can lead to increased soybean yield losses due to selection for evolved *P. sojae* pathotypes (Wrather and Koenning, 2006). More than 200 *P. sojae* pathotypes have already been isolated from soils, and selection for new ones seems to be most rapid when there is partial resistance to the deployed soybean varieties (Stewart et al., 2014). For *Pythium* species, major soybean resistance genes have not been identified, so seed treatments with pesticides remain the primary management strategy management tool to reduce seedling rot and damping off (Dorrance et al., 2009; Vossenkemper et al., 2015). Treatments include combinations of metalaxyl and mefenoxam, and fosetyl-Al which provide protection against many different broad host-range *Pythium* pathogens during seed germination (Esker and Conley, 2011). *Pythium* species vary in their sensitivity to these pesticides (Dorrance et al., 2004; Weiland et al., 2014), so other chemical or management practices are needed.

Many soil-derived *Pseudomonas* strains have been identified that are potentially useful biocontrol agents of oomycetes. These include *Pseudomonas putida* (Shi et al., 2013), *Pseudomonas fluorescens* 113 (Redondo-Nieto et al., 2013), *Pseudomonas* sp. SH-C52 (Van Der Voort et al., 2015), and *Pseudomonas chlororaphis* (Guyer et al., 2015). The diverse range of inhibitory compounds that pseudomonads produce (Lareen et al., 2016), includes volatiles (De Vrieze et al., 2015; Hunziker et al., 2015), siderophores (Matthijs et al., 2007), phenazines (Pierson and Pierson, 2010), antibiotics (Rode et al., 1989; Farrow and Pesci, 2007) and metabolites produced by non-ribosomal peptide synthetases (de Bruijn et al., 2007; Perneel et al., 2008; Loper et al., 2012; Raaijmakers and Mazzola, 2012). Pseudomonads persist in freshwater habitats (Morris et al., 2010; D’souza, 2013; Morris et al., 2013) and have been shown to inhibit oomycetes (De Vrieze et al., 2015; Novinscak et al., 2016), including a fish pathogen (Liu et al., 2015). Based on those observations, we hypothesized that pseudomonads in aquatic ecosystems, might be an untapped resource of unique biocontrol compounds that are effective against oomycete plant pathogens.

New approaches are needed to screen larger numbers of microbes for potential activity as biocontrol agents of oomycetes. Environmental isolates need to be identified with genetic markers that can be used to identify individuals. Variation in 16S sequences is not sufficient to provide information on intra-species variation. The genetic variation of housekeeping genes such as *gyrB, rpoB*, and *rpoD* can provide a higher level of resolution for the characterization of pseudomonads (Mulet et al., 2010). Identification of new biocontrol agents requires direct screening of isolates to assay for protection of plants against diverse pathogens, but these types of assays are not easily scaled up to handle many isolates. Additionally, limiting candidates to bacteria that directly protect plant roots from oomycete infection, excludes those organisms that synthesize antagonistic compounds, that could be produced industrially for application as new seed treatments (Kleigrewe et al., 2016). The chemical identification of novel antagonistic compounds is itself a challenging endeavor. A large-scale, citizen science initiative that has made use of undergraduate researchers at several universities to identify new antibiotics, has not to our knowledge lead to the identification of antimicrobial compounds (Davis et al., 2017). Direct sequencing of antagonistic strains may also not be useful alone, in identifying the operon responsible for antagonistic activity, since *Pseudomonas* sp. have large genomes, and as we have already noted, can produce a large number of inhibitory compounds.

The present study examines a collection of 330 *Pseudomonas* spp. (Chatterjee et al., 2017) for the expression of compounds antagonistic to pathogens of *Glycine max*. Originally collected from soil samples in Bowling Green, OH, and from the Central Basin of Lake Erie, this set has been divided into 13 distinct clades using sequence differences in the housekeeping gene *gyrB*. As described herein, 118 of the isolates exhibited antagonistic activity against one or more oomycete species, with a significant majority of the antagonistic strains having originated in the Lake Erie ecosystem.

Based on our assessment of the phylogenetic diversity, and the dissimilar antagonistic profiles of these isolates, we conclude that that these aquatic isolates produce a diverse set of compounds that inhibit the growth of oomycetes. To identify the molecular basis of the antagonistic activity in environmental pseudomonads, we initiated a strategy to optimize transposon mutagenesis in individual isolates to identify isolates with loss of antagonistic activity (Chatterjee et al., 2017; Davis et al., 2017). These isolates can then be sequenced to identify the operons responsible for the antagonistic activity. This strategy was first implemented for *Pseudomonas* strain 06C 126, which inhibits the growth of multiple human pathogens (Chatterjee et al., 2017) and also showed antagonistic activity to some oomycetes. Transposon mutagenesis of this isolate targeted an operon encoding a predicted siderophore. Under iron-limiting conditions, this strain inhibited the growth of four *Pythium* species, while transposon mutagenesis of this locus resulted in a loss of inhibitory activity. Taken as whole, we outline a bioassay to efficiently screen pseudomonads for antagonism against oomycetes, and provide a genetic strategy to select isolates for further experiments where these isolates can be evaluated as biocontrol agents, or as genetic reservoirs of operons that can be utilized to produce a new generation of seed treatments.

## Materials and Methods

### Culturing and Maintenance of Pseudomonads and Oomycete Strains

Environmental isolates were retrieved from the Lake Erie Central Basin, Station 880 (41°55′00″N, 81°31′00″W) in February 2012 and from soil in Bowling Green, OH on April 5th, 2012 (Chatterjee et al., 2017). Water samples were collected from the photic zone at a depth of one meter and a temperature of 1.5°C; soil samples were obtained from topsoil at a depth of one inch and a temperature of 17.0°C. All *Pseudomonas* strains were cultured in Nutrient Broth (NB) and on NB solid media (BD Difco) with 1.5% agar (BD Difco) and incubated at 23°C. *P. sojae* and *Pythium* species were cultured on V8 agar media (20% V8 juice, 0.25% CaCO_3,_ and 1.5% agar) and incubated at 23°C. *P. sojae* strain P6497 (Tyler et al., 2006) was obtained from the World Phytophthora and Oomycete Genetic Resource Collection, (Stredansky et al., 2000) and *Pythium* strains were isolated from diseased soybean plants, and were kindly provided by Ann Dorrance, Ohio State University.

### Phylogenetic Analysis of Pseudomonads

Phylogenetic analysis of pseudomonas isolates using primers targeting the *gyrB* gene have been previously described (Chatterjee et al., 2017). For population structure analysis, 659 bp of the *gyrB* gene were aligned and a neighbor-joining tree was constructed using Jukes-Cantor nucleotide distance measurement in CLC Main Workbench. iTOL was used to view the tree and overlay antagonistic data (Letunic and Bork, 2016).

### Antagonistic Assays

Antagonistic assays were performed by co-cultivation of individual *Pseudomonas* isolates with each *Pythium* species as well as with *P. sojae* strain P6497. For antagonistic assays involving *P. sojae*, zoospores were produced by repeated washing with sterile deionized water of rapidly growing hyphal cultures on V8 plates (Morris and Ward, 1992). The zoospore suspension was induced to encyst by vortexing for 30 s, and then poured onto 150 mm × 15 mm petri plates with V8 media. The cultures were incubated without further disturbance for 2 h at 23°C, and then the excess liquid was poured off, and the plates were dried in a sterile laminar flow hood overnight. Microscopic examination of the plates after overnight incubation revealed a lawn of hyphal germlings. A 96 pin replicator was used to transfer one microliter of an overnight culture of *Pseudomonas* onto the oomycete spread V8 agar plate. For antagonistic activity against *Pythium* species, assays were set up by arraying 3 mm plugs of *Pythium* cultures growing on V8 media onto a fresh V8 plate. Approximately 1 μl of an overnight *Pseudomonas* culture was then transferred to locations adjacent to the plugs using a multi-channel pipette. For all assays, antagonistic activity was recorded after 2 days at 23°C. Only strong inhibition, exhibited as inhibited growth >4 mm from the bacterial colony was scored as positive. All assays were performed in triplicates. A chi-square test was used to determine if there was more activity among soil or water-derived strains.

Antagonistic assays with *Pseudomonas* 06C 126 wildtype strain and transposon-derived *qbs* mutant was performed as using an iron-deficient medium of casamino acid agar medium (GCA, 0.5% w/v casamino acids, 0.118% w/v of anhydrous potassium phosphate, 0.25% w/v of magnesium sulfate heptahydrate, and 0.2% glucose). *Pythium* strains were cultured and maintained on V8 plates, as described above, and 3 mm plugs containing isolates were transferred to GCA plates. One microliter aliquots from overnight cultures of wildtype 0C6 126 or the *qbs* mutant were transferred between the plugs. Inhibition of hyphal growth around the bacterial colony was assessed after 3 days at 23°C.

### Strain 06C 126 Genome Sequencing, Annotation, and Heatmap

The sequence of wildtype strain 06C 126 has been deposited in NCBI under the accession number SAMN05727803 (Chatterjee et al., 2017). The genome was annotated by the Joint Genome Institute Genomes Online Database (GOLD) database (Liolios et al., 2006) and has been deposited under the submission ID 91354. The biosynthetic gene cluster (BGC) heatmap was generated using the JGI ABC database. The BGC ID #161755954 in strain 06C 126 was predicted to encode a secondary metabolite with antagonistic activities. Protein family (Pfam) similarity was determined using the Jaccard Index and modified Jaccard Index scores. The top 30 hits in the JGI ABC database were used to generate the heatmap. Only BGCs with three of more similar Pfams were included in the analysis.

## Results

**FIGURE 1:**
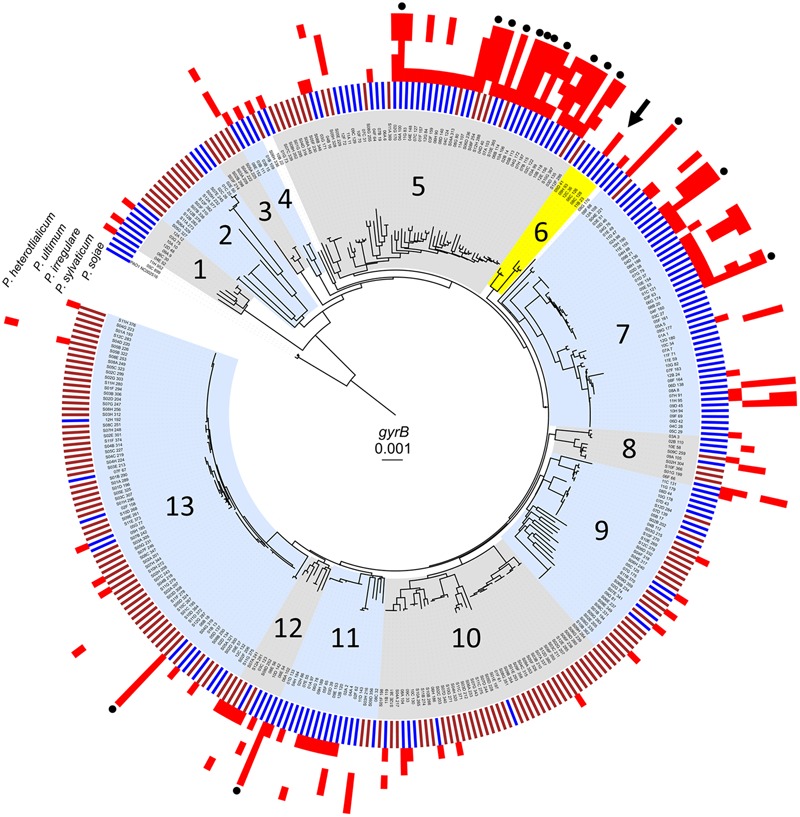
Population-level diversity and antagonistic activity among pseudomonads. Neighbor-joining phylogenetic tree based on partial sequence of the *gyrB* gene for 330 strains (Chatterjee et al., 2017), overlaid with data for habitat (inner bars: brown, soil; blue, water). 163 strains were isolated from the Central Basin of Lake Erie and 167 were from soil in Bowling Green, OH, United States. Antagonistic activity against *P. sojae, P. sylvaticum, P. irregular, P. ultimum*, and *P. heterothalicum* is indicated by a red block. Strains that exhibit activity against all oomycetes are identified by a black circle on the outermost edge of the tree. Highlighted in yellow is population 6 which includes strain 06C 126 (black arrow).

Water samples were collected from the photic zone at a depth of one meter and a temperature of 1.5°C; soil samples were obtained from topsoil at a depth of one inch and a temperature of 17.0°C (Chatterjee et al., 2017). We determined the population-level diversity of *Pseudomonas* strains isolated from the freshwater and soil habitats through neighbor-joining analysis of the *gyrB* housekeeping gene. This locus provides a higher level of intra-species resolution for environmental isolates than 16S sequences (Mulet et al., 2010). All strains were identified as *Pseudomonas* spp. based on homology to other *gyrB* sequences in the NCBI database. Each of 330 isolates was partitioned according to *gyrB* genotypic diversity to visualize the collective phylogenetic structure as shown in **Figure 1**. Thirteen phylogenetic groups, consisting of three or more strains were identified based on nucleotide divergence and branching patterns. To investigate ecotype, data pertaining to habitats were superimposed onto phylogeny to determine if certain environments were dominated by particular phylogenetic groups, suggesting adaptation to a particular niche (**Figure 1**, inner ring). Most groupings consisted of pseudomonads from soil or aquatic habitats. Isolates from phylogenetic groups 2, 3, 10, and 13 were soil-derived while phylogenetic groups 1, 4, 6, 7, and 11 consisted of isolates almost exclusively from water. Interestingly, Phylogenetic groups 5, 8, 9, and 12, contained isolates from both soil and aquatic environments. Phylogenetic diversity, based on the *gyrB* gene, was sufficient to distinguish most isolates.

### Environmental Pseudomonads Inhibit Pathogenic Oomycetes

**FIGURE 2:**
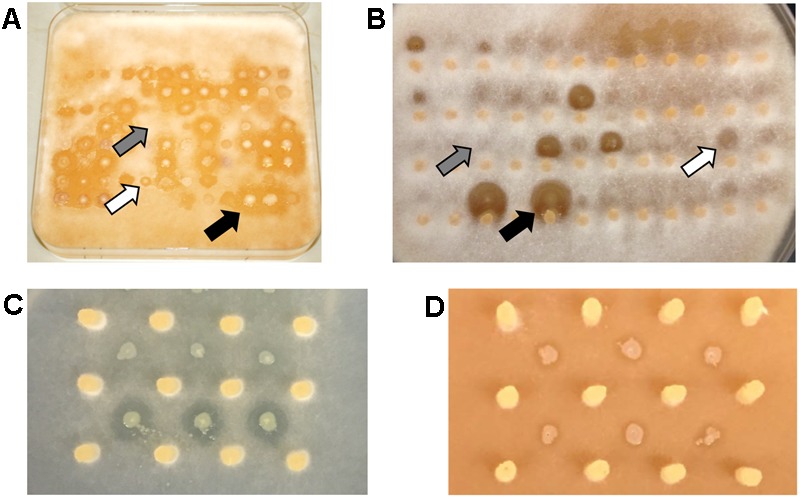
Antagonistic activity against oomycetes and loss of killing phenotype. Photograph of antagonistic assay of **(A)** 96 and **(B)** 60 water strains against *P. sojae* and *P. sylvaticum*, respectively. All bacterial isolates were competed against five oomycetes on V8 medium. Gray, white, and black arrows indicate no, weak, and strong antagonistic activity, respectively. **(C)** Wildtype strain 06C 126 (bottom row) and *qbs* mutant (top row) showing loss of antagonistic activity on GCA medium. A zone of clearing is observed with the wildtype strain and absent from the mutant. **(D)** Wildtype strain 06C 126 (bottom row) and transposon mutant (top row) exhibiting antagonistic activity on V8 medium. A zone of clearing is observed around the wildtype and mutant strains. Yellow plugs in **(B–D)** represent the source of *P. sylvaticum* inoculum.

All 330 strains were tested through interspecific competition against the five different plant pathogens resulting in 1,650 individual interactions. To assay for bacterial inhibition, of hyphal growth, environmental isolates were positioned adjacent to rapidly growing hyphae of oomycetes on V8 agar plates. After 2 days when the plates were scored, all of the environmental isolates formed robust colonies on this media which is typically used to maintain oomycete cultures (Erwin and Ribeiro, 1996). A zone of inhibition of at least 4 mm surrounding the environmental isolate was scored as strong antagonistic activity against the oomycete pathogen (**Figures 2A,B**). Microscopic examination of the clearing zone revealed that hyphae were largely excluded, and that some of the isolates were capable of swarming. Results showed that 118 out of 330 environmental isolates (36%) effectively inhibited at least one pathogen (**Figure 1**). Antagonistic isolates showed consistent levels of inhibition against oomycetes in replicate assays.

Antagonistic activity was observed from both water and soil strains and among all phylogenetic groups except 2 and 3 which consisted almost entirely of strains from soil (**Table 1**). Out of 118 antagonistic strains, 88 and 30 were water and soil-derived, respectively. Sixty-four strains exhibited specific interactions and inhibited only one pathogen, while 54 were more general in their activity and antagonized more than one oomycete (**Figure 1**). For instance, strains from phylogenetic groups 1, 4, and 9 all exhibit specific activity against one pathogen, while isolates in other phylogenetic groups exhibit both specific and a broader range of activity. In all, 253 antagonistic events were observed, and activity from water-derived strains was significant, 205 out of the 253 (chi-square test, *P* < 0.0001), compared to soil isolates. Inhibitory events were observed most frequently in phylogenetic groups 5 and 7 which is composed of more isolates compared to other phylogenetic groups and are mostly water-derived. Although population 13 had the most number of strains and were almost entirely from soil, only 15 antagonistic events were observed; 13 out of 15 occurred by soil-derived isolates (**Table 1**). Of particular interest among the 330 pseudomonads were 16 genetically diverse isolates that were able to inhibit all pathogens (**Figure 1**, black circles) meaning that the *Phytophthora* and four *Pythium* isolates were susceptible to the produced antagonistic compounds. All 16 strains were isolated from Lake Erie and 14 grouped within phylogenetic groups 5 and 7. Overall, *P. sojae* was the most susceptible and was inhibited by 103 bacterial strains (**Table 1**). All *Pythium* species were inhibited by less than half the antagonistic events compared to *P. sojae*; *P. sylvaticum* was the least susceptible, and was antagonized by only 31 strains. Taken together, significantly more pseudomonads from a freshwater habitat inhibit oomycetes compared to soil isolates. Moreover, their phylogenetic diversity and dissimilar antagonistic profiles suggest the production of unique compounds. These results support the concept that water-derived bacteria produce effective compounds that inhibit pathogens.

**Table 1:** Numbers of antagonistic events against oomycetes.

		Number of *Pseudomonas* strains that are antagonistic against an oomycete					
Population (number of strains)	^a^Habitat	^b^*P. soj*	^b^*P. syl*	^b^*P. irr*	^b^*P. ult*	^b^*P. het*	Total
1 (7)	7 water	3	–	–	–	–	3
	0 soil	–	–	–	–	–	–
2 (14)	3 water	–	–	–	–	–	–
	11 soil	–	–	–	–	–	–
3 (5)	0 water	–	–	–	–	–	–
	5 soil	–	1	1	–	1	3
4 (4)	4 water	2	–	–	–	–	2
	0 soil	–	–	–	–	–	–
5 (56)	38 water	29	12	13	18	18	90
	18 soil	6	5	3	3	4	21
^3^6 (6)	1 water	1	1	0	0	0	2
	0 soil	–	–	–	–	–	–
7 (49)	48 water	22	6	11	12	15	66
	1 soil	–	–	–	–	–	–
8 (9)	5 water	1	3	1	1	1	7
	4 soil	1	–	–	–	–	1
9 (34)	12 water	2	–	–	–	–	2
	22 soil	4	–	–	–	–	4
10 (42)	6 water	4	1	1	–	–	6
	36 soil	2	–	–	–	–	2
11 (18)	16 water	9	–	2	2	2	15
	2 soil	–	–	–	–	–	–
12 (8)	5 water	3	1	2	1	2	9
	3 soil	3	–	–	–	–	3
13 (75)	11 water	2	–	–	–	–	2
	64 soil	7	1	1	1	3	13
Undefined (3)	2 water	1	–	–	–	–	1
	1 soil	1	–	–	–	–	1

Total (330)	Water	79	25	30	34	38	205
	Soil	24	7	5	4	8	48
	Total	103	35	35	38	46	253

*^*a*^Number of strains in a habitat.*^*b*^*P. soj, P. sojae*; *P. syl, P. sylvaticum*; *P. irr, P. irregular*; *P. ult, P. ultimum*; *P. het, P. heterothalicum.*

### Antagonistic Activity of Strain 06C 126 and the *qbs* Biosynthetic Gene Cluster (BGC)

**FIGURE 3:**
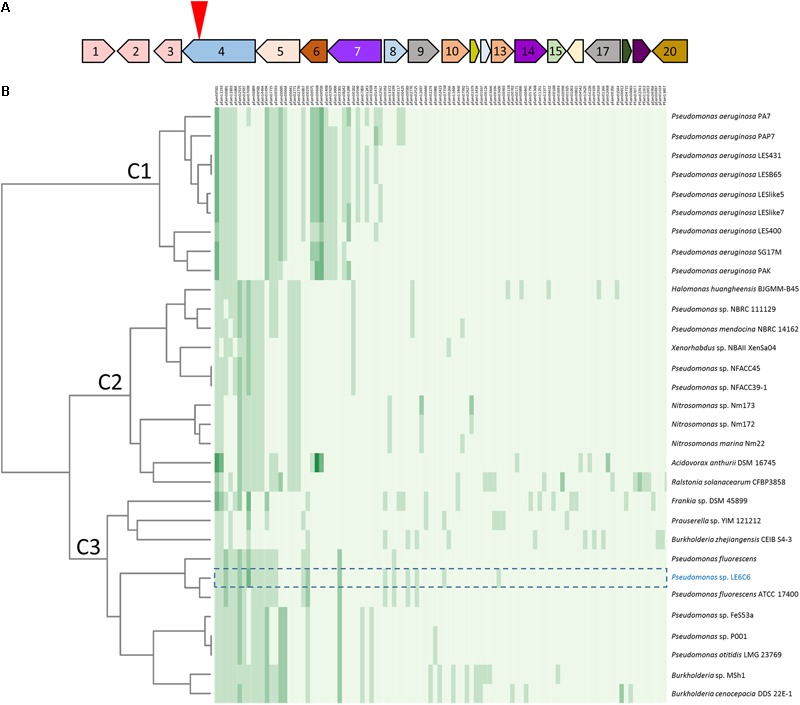
Pfam similarity to the 06C 126 *qbs* gene cluster. **(A)** Strain 06C 126 *qbs* BGC is predicted to encode a low affinity siderophore. Numbers in open reading frames (ORFs) correspond to ORFs in **Table 2**. The inverted red triangle indicates the site of transposon insertion. Same-colored ORFs represent identical Pfams. **(B)** Heat map of BGCs with similar Pfams to *qbs* BGC. Strain 06C 126 is highlighted in blue, and the BGC is outlined with a dashed line. Clade C3 represents BGCs with the most similar Pfams to the 06C 126 BGC. Numbers listed on the *X*-axis (top) correspond to Pfams in JGI ABC; predicted proteins and Pfams for strain 06C 126 BGC are in **Table 2**. The phylogeny on the *Y*-axis (left) was determined by both Pfam similarity and number of Pfams in a BGC from the most similar gene clusters in JGI ABC. More than one Pfam of the same type can be found in a BGC and is indicated by green shading and represent 0, 1, 2, 3, 4, and 5 Pfams. Bacterial strains are listed on the right *Y*-axis.

Transposon mutagenesis was first implemented in one of these environmental isolates to identify the molecular basis of antagonism against pathogenic isolates taken from the lungs of cystic fibrosis patients (Chatterjee et al., 2017). Sequencing of the 06C 126 mutant confirmed the presence of a single transposon insertion in the bacterial genome so that the phenotype is unequivocally linked to this locus. BLAST analysis of the mutated operon identified a 14.8 kb *qbs* locus in the water-derived strain 06C 126 that was 99% similar to a locus in *Pseudomonas fluorescens* ATCC 17400 (**Figure 3A**, ORRs 4–15). This locus is predicted to encode a siderophore in *P. fluorescens* and was found to inhibit the growth of *Pythium debaryanum* (Matthijs et al., 2007). Because of the *qbs* locus similarity between 06C 126 and *P. fluorescens* ATCC 17400, we hypothesized that the wildtype strain 06C 126 might also be capable of inhibiting oomycete pathogens through competition involving iron acquisition by siderophores. We repeated our antagonistic assays with the wildtype 06C 126 strain and the transposon-derived *qbs* mutant, on iron-limited GCA medium. Differential antagonistic activity was observed from the *qbs* mutant on GCA and V8 media (**Figures 2C,D**). Under iron limiting conditions, the mutant lost the ability to inhibit *Pythium* spp. Conversely, antagonism was observed against only *P. sojae* and *P. sylvaticum* on nutrient rich medium (**Figure 2**, black arrow) suggesting dissimilar inhibitory compounds are produced under different nutritional conditions (**Figures 2C,D**). *P sojae* did not grow well enough on GCA media to enable testing for inhibition under iron limiting conditions.

To identify if other bacteria, besides 06C 126 and ATCC 17400, encode the *qbs* locus, we utilized the JGI Atlas of Biosynthetic Gene Cluster (ABC) (Hadjithomas et al., 2017) to search for homologous gene regions among other bacteria. The JGI ABC database contains over 60,000 bacterial genomes that encode more than 1.1 million biosynthetic gene clusters (BGCs). BGCs are a set of genes predicted to encode a secondary metabolite and found within close proximity of each other. JGI ABC identified the *qbs* locus within a BGC (ID #161755954) comprised of 20 open reading frames (ORFs), one of which the transposon disrupted (**Figure 3A** and **Table 2**). To determine if this gene cluster is frequent among genomes within the JGI ABC database, we generated a heatmap of the 30 most similar hits of the predicted protein families (Pfam) to the *qbs* BGC (**Figure 3B** and **Table 2**). Three clades were generated in the analysis (C1–C3, **Figure 3B**). C1 consists entirely of BGCs from nine *Pseudomonas aeruginosa* strains which are most dissimilar from the BGC in strain 06C126; C2 consist of 11 strains from six genera, and C1 comprises of the most similar BGCs and consists 11 strains from four genera. The 06C 126 BGC consists of 27 Pfams. Results validated the NCBI BLAST search showing that the locus is most similar to *P. fluorescens* ATCC 17400, and the other closely related BGC was also from a *P. fluorescens* strain; however, the predicted Pfams were not all identical to the ones in the 06C 126 BGC. These three strains were similar in Pfams 1–7 in the cluster (00501, 13193, 00891, 12833, 16864, 12515, 02597 in **Figure 3B** and **Table 2**), 9–14 (00899, 00581, 14464, 10494, 07715, and 00593), 20 and 21 (00587 and 00155), 28 (03301), and 39 (13372). The remaining 10 Pfams were either unique to strain 06C 126 or shared with one other *P. fluorescens* strain. No other BGCs were as similar in Pfam content. Results from the NCBI nucleotide BLAST analysis and a search for homologous regions in JGI ABC, suggest the *qbs* BGC is rare in these databases.

**Table 2:** Strain 06C 126 *qbs* gene cluster.

ORF	^a^Gene ID	Gene	AA length	Protein	^a^Pfam	^b^Best hit genome
1	2686041894	–	418	Major Facilitator Superfamily (MFS)	pfam07690 MFS	*Delftia acidovorans* Cs1-4
2	2686041895	–	447	H+ symporter	pfam07690 MFS	*Streptomyces* sp. AA4
3	2686041896	–	404	Cyanate transporter	pfam07690 MFS	*Rhodococcus equi* ATCC 33707
4	2686041897	*qbsL*	905	AMP dependent synthetase	pfam13193 AMP-binding enzyme; pfam16864 Dimerisation domain; pfam00891 O-methyltransferase; pfam00501 AMP-binding enzyme	*Hahella chejuensis* KCTC 2396
5	2686041898	*qbsK*	600	Acyl-CoA transferase	pfam02515 CoA-transferase family III	*Ralstonia solanacearum* IBSBF1503
6	2686041899	*qbsJ*	345	Methyltransferase	pfam00891 *O*-methyltransferase	*Acidovorax avenae citrulli* AAC00-1
7	2686041900	*qbsI*	669	TonB-dependent receptor	pfam07715 and pfam00593 TonB-dependent Receptor	*Acidovorax avenae citrulli* AAC00-1
8	2686041901	*qbsA*	318	AraC family transcriptional regulator	pfam12833 Helix-turn-helix domain	*Pseudomonas protegens* Pf-5
9	2686041902	*qbsB*	363	Kynurenine transaminase	pfam00155 Aminotransferase class I and II	*Stenotrophomonas maltophilia* K279a
10	2686041903	*qbsC*	387	Molybdopterin converting factor	pfam00581 Rhodanese-like domain; pfam00899 ThiF family	*Hahella chejuensis* KCTC 2396
11	2686041904	*qbsD*	137	Peptidase	pfam14464 Prokaryotic homologs of the JAB domain	*Thermomonospora curvata* DSM 43183
12	2686041905	*qbsE*	90	Molybdopterin synthase	pfam02597 ThiS family	*Hahella chejuensis* KCTC 2396
13	2686041906	*qbsF*	284	Tryptophan 2,3-dioxygenase	pfam03301 Tryptophan 2,3-dioxygenase	*Meiothermus ruber* 21, DSM 1279
14	2686041907	*qbsG*	461	Kynurenine 3-monoxygenase	pfam01494 FAD binding domain	*Pseudoxanthomonas suwonensis* 11-1
15	2686041908	*qbsH*	218	Kynurenine formamidase	pfam04199 Putative cyclase	*Nocardioidaceae bacterium* Broad-1
16	2686041909	–	172	Nicotinamidase	pfam00857 Isochorismatase family	*Pseudomonas brassicacearum brassicacearum* NFM421
17	2686041910	–	482	Transcriptional regulator	pfam00392 Bacterial regulatory proteins, gntR family; pfam00155 Aminotransferase class I and II	*Pseudomonas protegens* Pf-5
18	2686041911	–	148	Acetyltransferase	pfam00583 Acetyltransferase (GNAT) family	*Pseudomonas protegens* Pf-5
19	2686041912	–	223	NADH-azoreductase	pfam02525 Flavodoxin-like fold	*Pseudomonas protegens* Pf-5
20	2686041913	–	450	Transporter	pfam07158 Dicarboxylate carrier protein MatC N-terminus; pfam03600 Citrate transporter	*Agrobacterium radiobacter* K84

*^*a*^Gene ID and Pfam numbers refer to the JGI database. ^*b*^Best hit genome results are from a bi-directional orthologous search of the ORF.*

To identify other possible products involved in differential activity observed on nutrient rich and poor media (**Figures 2C,D**), the genome of 06C 126 was analyzed for other BGCs that may encode secondary metabolites. Twenty-one other BGCs were identified (**Table 3**). Of these, five BGCs were predicted to encode a non-ribosomal peptide synthetase (NRPS) and two to encode a bacteriocin. Both types of operons are diverse in nature and are known to produce metabolites that are inhibitory to pathogens (Felnagle et al., 2008; Al-Mathkhury et al., 2011; Han et al., 2014). The remaining 14 BGCS are putative, and of unknown function. Our results suggest the water-derived strain 06C 126, encodes at least two products that have the ability to inhibit oomycetes, and supports the concept that water derived bacteria express secondary metabolites that are effective against pathogens.

**Table 3:** Biosynthetic gene cluster (BGCs) identified in strain 06C 126.

^a^Cluster ID	Gene count	^a^Scaffold	Start coordinates	End coordinates	BC length	Predicted product
161755951	43	2684657017	167735	226276	58542	nrps
161755952	12	2684657029	1641540	1662558	21019	Putative
161755953	32	2684657029	1882486	1924261	41776	Putative
161755954	20	2684657029	2172318	2195724	23407	Putative
161755955	13	2684657029	2308191	2326458	18268	Putative
161755956	17	2684657029	2519957	2551442	31486	Putative
161755957	10	2684657029	2786121	2796966	10846	Bacteriocin
161755958	17	2684657033	53641	74271	20631	Putative
161755959	6	2684657033	828789	836574	7786	Putative
161755960	7	2684657033	1056373	1068783	12411	Putative
161755961	13	2684657033	1286290	1302851	16562	Putative
161755962	3	2684657018	1	10330	10330	nrps
161755963	8	2684657033	1370827	1379691	8865	Putative
161755964	25	2684657033	1470770	1523714	52945	nrps
161755965	6	2684657033	1872795	1879961	7167	Putative
161755966	11	2684657023	297527	308408	10882	Bacteriocin
161755967	40	2684657023	540632	602020	61389	nrps
161755968	27	2684657029	290832	334221	43390	Putative
161755969	6	2684657029	406619	415350	8732	Putative
161755970	5	2684657029	1264493	1269762	5270	Putative
161755971	33	2684657029	1418103	1462110	44008	nrps
161755972	8	2684657029	1503277	1509690	6414	Putative

*^*a*^Cluster ID and scaffold coordinates are those of the JGI IGM/M database.*

## Discussion

This study demonstrated the ability of selected environmental strains to inhibit the hyphal growth of *P. sojae*, and four *Pythium* species under conditions which enabled rapid growth of the oomycete pathogens. Prior screening studies to identify novel oomycete biocontrol agents have made use of only soil or rhizosphere-derived isolates. In earlier work, we observed that transcriptome analysis of a Lake Erie microbial community revealed the presence of genes from several oomycete species (Edgar et al., 2016) suggesting that *Pseudomonas* and oomycete strains co-exist in this habitat and likely compete for resources. Based on these predicted interactions, and observations that potent inhibitory compounds have been isolated from strains within water habitats (Majik et al., 2014; Su et al., 2014; Igumnova et al., 2016), we sought to compare the effects of soil- and water-derived pseudomonads against oomycete soybean pathogens using a combined approach involving experimentation of culturable strains, and whole genome analysis. Significantly there were more aquatic than soil isolates that were antagonistic to at least one oomycete species, and all 16 isolates that were antagonistic to all five oomycetes, were isolated from Lake Erie.

Whole genome sequencing of pseudomonads with potential utility as biocontrol agents has confirmed that individual strains may produce a diverse repertoire of antimicrobial compounds (Baltrus et al., 2011; Loper et al., 2012; Redondo-Nieto et al., 2013; Van Der Voort et al., 2015). For example *Pseudomonas* sp. SH-C52 which is antagonistic to *Rhizoctonia solani* and the late blight pathogen *Phytophthora infestans* contains NRPSs which produce the antifungal peptides thanamycin, thanapeptin, brabantamide, and three other incompletely characterized compounds. Sequencing of 10 strains classified as from the *Pseudomonas fluorescens* group, revealed that the common core genome across all strains included only 45–52% of the total number of genes (Loper et al., 2012). A similar range of genomic diversity was revealed by sequencing 19 phytopathogenic isolates of *Pseudomonas syringae* (Baltrus et al., 2011). Although these genome-based studies strongly suggest pseudomonads produce inhibitory compounds, direct experimentation is necessary to identify genes involved in antagonistic activity.

We utilize a bacterial population-level approach involving phylogenetics, whole genome sequencing, and experimentation to directly identify genes involved in a particular function. This methodology has been applied to study the population structure and functional relationships among *Vibrio* strains associated with virulent trait diversity (Wildschutte et al., 2010), marine habitat association (Preheim et al., 2011), and competition among environmental *Vibrio* strains (Cordero et al., 2012). A similar population-level approach was used with pseudomonads to identify strains that inhibit human pathogens isolated from cystic fibrosis patients (Chatterjee et al., 2017). In the current survey, we sought to identify if this collection of strains, particularly water-derived isolates, can effectively inhibit a different phylogenic group of plant pathogens. Phylogeny was overlaid with habitat and antagonistic activity of individual pseudomonads against five oomycete, to resolve structure and antagonistic functional relationships. Genetic diversity was observed within and between phylogenetic groups and among both soil and water-derived strains. However, significantly more freshwater ecotypes exhibited activity against oomycetes. Two phylogenetic groups (phylogenetic groups 5 and 7; **Figure 1** and **Table 1**) composed mostly of water-derived strains, had 14 isolates capable of antagonizing all five oomycete pathogens. Twenty-five freshwater strains inhibited *P. sylvaticum* which was shown to be the cause of most soybean oomycete infections in the survey of 2011–2012 (Rojas et al., 2017). Notably, these pseudomonads were isolated from a cold freshwater habitat, and cold temperatures are a risk factor for seedling losses due to oomycete infections. Environmental pressures in this lake system might select for active antagonistic compounds that are effective biocontrol agents. Ecotype diversity with variable antagonistic profiles suggest these strains exhibit a variety of antagonistic factors that are effective against devastating oomycetes.

We have shown that antagonistic activity, coupled with transposon mutagenesis, are reliable methods to identify the molecular basis of inhibition among environmental isolates (Cordero et al., 2012; Chatterjee et al., 2017; Davis et al., 2017). On iron deficient medium, the wildtype strains 06C 126 effectively inhibited all *Pythium* species while the *qbs* mutant lost this antagonistic activity (**Figure 2C**). Although *P. fluorescens* ATCC 17400 exhibits 99% homology with ORFs 4–15 (Chatterjee et al., 2017), eight additional genes are in the 06C 126 BGC and these may further modify the predicted siderophore. Heatmap results suggest this BGC is rare in the JGI ABC database (**Figure 3B**). Although two *P. fluorescens* strains show close similarity in Pfam content, more than 1.1 million BGCs were searched in the analysis. A bi-directional ORF search was also performed to identify additional similar genes surrounding this locus; results showed that surrounding gene regions of 14 ORFs are most closely related to loci in other *non-Pseudomonas* strains (**Table 2**) suggesting genomic variability. It is notable that this BGC encodes a unique siderophore that provides a fitness advantage in a freshwater ecosystem, such as Lake Erie where iron can be a limited resource (North et al., 2007; Davis et al., 2015), and may be involved in antagonistic activity through competition for iron.

The observed differential activity of wildtype strain 06C 126 and the *qbs* mutant on nutrient-rich and iron-deficient media suggest that distinct genomic regions, other than the *qbs* locus, may contribute to the inhibition of these oomycete species under different nutrient conditions. The differential regulation of secondary metabolites under diverse conditions has been well studied. For instance, strains of actinomycetes have been shown to exhibit distinct antimicrobial activities under different temperature and pH conditions (Basilio et al., 2003), nitrogen and carbon sources have been found to affect iturin A antibiotic production by *Bacillus subtilis* (Iwase et al., 2009), and metabolite production by *Streptomyces coelicolor* is influenced, in some cases, by neighboring strains (Traxler et al., 2013). Thus, it would be unlikely that all products encoded by the 22 BGCs (**Table 3**) would be produced under identical conditions. Not only are these complex metabolites costly to produce, but it is unlikely that metabolites with different functions are expressed under the similar conditions. In strain 06C 126, we identified 21 additional BGCs with potential for antagonistic activity, suggesting this strain may produce distinct antagonistic compounds under certain environmental conditions.

We have already shown that transposon mutagenesis can be applied to some environmental strains of *Pseudomonads*, and combined with high throughput screening to identify the molecular basis of antagonism (Chatterjee et al., 2017; Davis et al., 2017). Once these techniques have been optimized for strains that inhibit oomycetes, high throughput screening can be implemented to identify the gene clusters that are responsible for the antagonistic activity against oomycete plant pathogens. These gene clusters can potentially be mobilized to other biocontrol strains, or transferred to strains that are optimized for antibiotic production.

## Author Contributions

AW and SN helped to design experiments and performed the antagonistic assays. PC characterized the *Pseudomonas* isolates and analyzed the data. PM and HW designed the experiments, and wrote the manuscript with contributions from AW, SN, and PC.

## Conflict of Interest Statement

The authors declare that the research was conducted in the absence of any commercial or financial relationships that could be construed as a potential conflict of interest.
